# An efficient overexpression method for studying genes in *Ricinus* that transport vectorized agrochemicals

**DOI:** 10.1186/s13007-022-00842-w

**Published:** 2022-01-26

**Authors:** Yongxin Xiao, Jinying Zhang, Yiting Li, Tom Hsiang, Xingping Zhang, Yongxing Zhu, Xiaoying Du, Junliang Yin, Junkai Li

**Affiliations:** 1 Hubei Key Laboratory of Waterlogging Disaster and Agricultural Use of Wetland/Institute of Pesticides/College of Agriculture/College of Life Science/College of Horticulture and Gardening, Yangtze University, Jingzhou, 434025 Hubei China; 2 School of Environmental Sciences, University of Guelph, Guelph, ON N1G 2W1 Canada

**Keywords:** l-Val-PCA (l-valine-phenazine-1-carboxylic acid conjugate), Vacuum infiltration, Subcellular localization, Plasma-membrane transporter, Recognizing and phloem loading

## Abstract

**Background:**

Plant plasma membrane transporters play essential roles during the translocation of vectorized agrochemicals. Therefore, transporters associated with phloem loading of vectorized agrochemicals have drawn increasing attention. As a model system, castor bean (*Ricinus communis* L.) has been widely used to detect the phloem mobility of agrochemicals. However, there is still a lack of an efficient protocol for the *Ricinus* seedling model system that can be directly used to investigate the recognition and phloem loading functions of plasmalemma transporters toward vectorized agrochemicals.

**Results:**

Here, using vacuum infiltration strategy, we overexpressed the coding gene for enhanced green fluorescent protein (eGFP) in *R. communis* seedlings by *Agrobacterium tumefaciens*-mediated transformation system. Strong fluorescence signals were observed in leaves, demonstrating that exogenous genes can be successfully overexpressed in seedlings. Subsequently, gene expression time and vacuum infiltration parameters were optimized. Observation of fluorescence and qRT-PCR analysis showed that eGFP strength and expression level reached a peak at 72 h after overexpression in seedlings. Parameter optimization showed *Agrobacterium* concentration at OD_600_ = 1.2, and infiltration for 20 min (0.09 MPa), return to atmospheric pressure, and then infiltration for another 20 min, were the suitable transformation conditions. To test the application of vacuum agroinfiltration in directly examining the loading functions of plasma membrane transporters to vectorized agrochemicals in seedlings, two LHT (lysine/histidine transporter) genes, *RcLHT1* and *RcLHT7,* were overexpressed. Subcellular localization showed the strong fluorescent signals of the fusion proteins RcLHT1-eGFP and RcLHT7-eGFP were observed on the cell membrane of mesophyll cells, and their relative expression levels determined by qRT-PCR were up-regulated 47- and 52-fold, respectively. Furthermore, the concentrations of l-Val-PCA (l-valine-phenazine-1-carboxylic acid conjugate) in phloem sap collected from seedling sieve tubes were significantly increased 1.9- and 2.3-fold after overexpression of *RcLHT1* and *RcLHT7*, respectively, implying their roles in recognition and phloem loading of l-Val-PCA.

**Conclusions:**

We successfully constructed a transient expression system in *Ricinus* seedlings and laid the foundation for researchers to directly investigate the loading functions of plasma membrane transporters to vectorized agrochemicals in the *Ricinus* system.

## Background

Vectorized agrochemicals are capable of increased systemic activity over the parent pesticides and thus, improve the efficacy of pesticides against insect pests and fungal pathogens, and allow for decreasing rates or usage and hence reduction of threats to the environment [1]. Some non-phloem-mobile parental compounds can be modified to be vectorized agrochemicals and acquire phloem translocation by conjugation with nutrients such as sugars or amino acids, after which the plasma membrane transporters will recognize those nutrients moieties and load the conjugated compounds into sieve tubes for transport in the phloem. Consequently, rational utilization of the plant membrane transporters has become a critical point for the design and syntheses of vectorized agrochemicals [2–4]. For example, the insecticidal fipronil derivatives [5], rotenone derivatives [6], and chlorantraniliprole derivatives [7] acquired phloem mobility by conjugation of active ingredients with specific amino acids. PCA, a natural product isolated from the metabolites of the phytopathogens *Pseudomonas* sp. M18, has been found to be effective against numerous soilborne fungal pathogens and registered as biofungicide against rice sheath blight in China [8]. Recently, a derivative PCA has achieved phloem translocation by conjugation of its carboxyl group with amino groups of L type amino acids [9, 10]. Among PCA derivatives, l-Val-PCA (l-valine-phenazine-1-carboxylic acid) exhibited the highest phloem mobility [9]. Since valine is a neutral amino acid and LHTs (lysine and histidine transporters) are classified as higher affinity transporters for neutral and acidic amino acids [11, 12], we speculated that LHTs could be potentially involved in the recognition and loading of l-Val-PCA.

Deciphering the recognition and loading functions of transporters toward vectorized agrochemicals can facilitate the rational utilization of these plasma-membrane carriers in modification strategies for non-phloem-mobile parental compounds. Previously, several systems have been used to investigated the recognition and loading functions of transporters toward vectorized agrochemicals. For example, *Xenopus laevis* oocyte was used to study the function of plant monosaccharide transporter RcSTP1 and reveal its affinity for a glucose-fipronil conjugate (GTF) [13]. *Arabidopsis thaliana* was used to detect the uptake ability of roots for an alanine-chlorantraniliprole conjugate after overexpressing *AtAAP1* [14]. Although above systems were suitable for gene heterologous expression, they are not designed to specifically survey the phloem mobility of vectorized agrochemicals. The *Xenopus laevis* oocyte system can be used to demonstrate the active transport of xenobiotics, but it can’t be used to specifically examine phloem mobility because the *Xenopus laevis* oocyte is animal cell, which does not have a vascular bundle. The *Arabidopsis thaliana* was limited by the difficulty of qualitative and quantitative manipulation, incubation of the leaf veins because of the cuticle, and the storage compounds in the leaf “buffered” most of the experimental treatments. Therefore, *Arabidopsis* exhibits few “phloem bleeding” upon incision of the bark [15]. The *Ricinus* seedling has been frequently used as a model plant system to qualitatively and quantitatively detect the phloem mobility of various agrochemicals, which has advantages in the analysis of sieve-tube sap with mature incubation method and thus possesses the property of investigating the response of the phloem loading system to pesticides in the leaf. Because the cotyledons of seedling without a cuticle, it can readily respond to incubation with solutes. Furthermore, *Ricinus* is one of the industrially important oilseed crops. Wilt disease caused by *Fusarium oxysporum* f. sp. *ricini* is an important oil and seed borne disease in castor and results in significant yield losses [16]. Thus, studies of phloem mobility of vectorized agrochemicals contribute to the control of castor wilt disease by foliar spraying. However, there is still a lack of an efficient method in seedlings that can be directly used to investigate the functions of plasma membrane transporters for recognition and phloem mobility of vectorized agrochemicals.

The *Agrobacterium tumefaciens*-mediated transformation system (ATMTs) is widely used for exogenous gene function analysis in plant. For example, ATMTs has been used to introduce a miraculin gene into callus of carrot (*Daucus carota* L.), and during cell division, the miraculin gene was highly expressed in transgenic callus lines [17]. In addition, ATMTs was also used to transfect the sections of young cotyledon in tomato (*Solanum lycopersicum* L.) to examine overexpression of target genes [18]. Furthermore, vacuum infiltration, widely described to enhance *Agrobacterium* infection, has been successfully used to produce transgenic plants in wheat [19], *Arabidopsis* [20], cotton [21], bean [22], banana [23], coffee [24], citrus [25], and watermelon [26]. This vacuum agroinfiltration protocol improves the transformation efficiency by enhancing the penetration of *Agrobacterium* into target tissues [27].

The transformation efficiency of an exogenous gene with transient expression was influenced by multiple parameters when mediated by vacuum agroinfiltration. For instance, Amoah et al. [28] found that increasing the *Agrobacterium* cell density, the duration of inoculation, and the vacuum pressure were able to enhance the expression of *uidA* when transforming inflorescent tissue in immature wheat. Accordingly, in the current study, parameters to be tested included the gene expression time for surveying fluorescence signal after infiltration, *Agrobacterium* cell density, and vacuum infiltration time, to optimize them for transformation to create an overexpression protocol for seedlings. After transformation *RcLHT1* and *RcLHT7*, the phloem mobility of vectorized agrochemical l-Val-PCA was assessed by HPLC.

## Materials and methods

### Plant materials

To develop a gene transient expression protocol for seedlings, seeds purchased from the Zibo Academy of Agricultural Science were sown into vermiculite following Rocher et al. [29]. Six days later, uniformly growing seedlings, with stem widths of 3.0 ± 0.2 cm, were selected, and the endosperm (20 mm length) was carefully removed for further experiments.

### Construction of vacuum agroinfiltration in *Ricinus* seedlings

To demonstrate that exogenous genes can be transiently expressed in seedlings, the eGFP gene was selected because it can acquire transient overexpression with the cauliflower mosaic virus (CaMV) 35S promoter [30]. The plant binary expression vector pART27-eGFP (College of Agriculture, Yangtze University) was transformed into *Agrobacterium tumefaciens* strain GV3101 by the freeze-melt method [31]. Then, GV3101 carrying pART27-eGFP was cultured for 20 h in 400 mL LB medium containing 50 μg/mL spectinomycin and 20 μg/mL rifampim. After centrifugation (Avanti JXN-30, Beckman Coulter, California, USA) at 8000*g* for 5 min, the collected *Agrobacterium* cells were resuspended in 200 mL buffer solution containing 10 mM MES (pH 5.6), 10 mM MgCl_2_ and 200 μM acetosyringone, to reach an OD_600_ of *Agrobacterium* cells of 1.0 [32].

Then endosperm-excised seedlings were soaked in the *Agrobacterium* suspension and subjected to vacuum infiltration (JX820D-1, SMAF, Shanghai, China) at 0.09 MPa for 20 min. When infiltration was complete, seedlings were transferred into Hogland solution and cultured in a cold-light source incubator (GDX-330, Safu, Ningbo, China) for 72 h at 18 °C in the dark [33]. Then the seedlings were observed under UV light (UVP BLAK-RAY B-100AP LAMP, Analytic Jena, Jena, Germany) to confirm eGFP expression.

### Optimization of overexpression time of exogenous gene

The eGFP signal was monitored at 100× magnification by laser confocal microscopy (TCS-SP8, Leica, Wetzlar, Germany) at 24 h, 48 h, 72 h, and 96 h, and then photographed. Because the wild type seedlings did not express *eGFP*, the relative expression level of *eGFP* after incubation for 2 h in wild type seedlings was used as the baseline control. For each treatment, cotyledons of at least three seedlings were harvested, quickly frozen in liquid nitrogen, and stored at − 80 °C for RNA isolation. The total RNA from *Ricinus* cotyledons was extracted using Total RNA Extraction Reagent (Vazyme, Nanjing, China) following a reported method [34]. The quality of RNA was measured with an UV–Vis spectrophotometer (Q6000M, Quawell, San Jose, USA).

For qRT-PCR analysis, the HiScript^®^ II First Strand cDNA Synthesis Kit (Vazyme, Nanjing, China) was used to synthesize the first-strand cDNA using 1 µg total RNA. Each 20 µL reaction contained 10 µL 2 × RT Mix, 4 µL HiScript II Enzyme Mix, 1 µL oligo (dT)_23_VN (50 µM), 1 µL random hexamers (50 ng/µL), and nuclease-free H_2_O up to 20 µL. The reverse transcription program was 25 °C for 5 min, 50 °C for 15 min, and 85 °C for 2 min.

The qPCR was performed in a CFX Connect™ Real-Time PCR detection system (Bio-Rad, California, USA). Each 20 µL reaction contained 10 µL 2 × ChamQ Universal SYBR qPCR Master Mix (Vazyme, Nanjing, China), 0.4 µL forward/reverse primers (10 µM), 1 µL cDNA, and nuclease-free H_2_O up to 20 µL. The *R. communis Actin* gene was used as the reference [13]. The relative expression levels of *eGFP* were calculated by the 2^−∆∆Ct^ method [35]. Significant treatment effects were assessed with ANOVA followed by mean separation using Dunnett’s test in SPSS software. Forward/Reverse primers of *eGFP* gene were designed using Primer Premier 5.0 software (Premier Biosoft International, Computing Associates, Palo Alto, USA) [36].

### Optimization of vacuum infiltration parameters

The parameters of vacuum infiltration were optimized by surveying eGFP signal strength. A series of *Agrobacterium* cell densities was used to infiltrate seedlings, and OD_600_ values at 0.4, 0.8, 1.2, and 1.6 were used. Different infiltration times were also tested, including 10 min, 20 min, 20 plus 20 min, and 40 min. The 20 plus 20 min treatment referred to vacuum infiltration for 20 min, then return to atmospheric pressure (about 2 min), and then infiltration for another 20 min. After 72 h, eGFP signal strength was observed at 100× magnification by laser confocal microscopy, and relative expression rate of eGFP was assessed by counting the cells with and without eGFP signal [37].

### Subcellular localization

To determine the expression and localization of the two transporters, RcLHT1 and RcLHT7, recombinants pART27-RcLHT1-eGFP and pART27-RcLHT7-eGFP were constructed as follows. CDS of RcLHT1 and RcLHT7 were downloaded from the NCBI database. Then two primers for full-length amplification of RcLHT1 and RcLHT7 were designed using Primer Premier 5.0 software. The 5′ end of the forward primer contained an Xho I site and 15 bp homologous sequences, which were located upstream of the Xho I site of pART27-eGFP. The 5′ end of the reverse primer also contained an Xho I site and 15 bp homologous sequences downstream. Phanta^®^ Max Super-Fidelity DNA Polymerase (Vazyme, Nanjing, China) was used for full-length amplification of *RcLHT1* and *RcLHT7* by PCR. Moreover, the empty vector pART27-eGFP was digested with Xho I for plasmid linear. Then PCR products and linear pART27-eGFP were separated and purified by 1% agarose gel electrophoresis. Subsequently, ClonExpress^®^ II One Step Cloning Kit (Vazyme, Nanjing, China) was used to connect target fragments to pART27-eGFP by the In-Fusion method [38]. Plant binary expression vector pCAMBIA1300-35S-PM-mCherry (MiaolingBio, Wuhan, China) was used as plasma membrane marker. Furthermore, marker and two constructed recombinant plasmids were transformed into *A. tumefaciens* strain GV3101 by the freeze-melt method. The transformant carrying two recombinant vectors was cultured for 18 h in 5 mL LB medium containing 50 μg/mL spectinomycin and 20 μg/mL rifampin. But the difference was that the transformant carrying marker was cultured in LB medium containing 50 μg/mL kanamycin. After centrifugation at 5000*g* for 5 min, the collected *Agrobacterium* cells were resuspended in MES solution containing 200 μM acetosyringone, to reach an OD_600_ of *Agrobacterium* cells of 0.4. The resuspension carrying marker was mixed with the resuspension carrying two recombinants in 50 mL centrifuge tubes following the ratio of 1:1, respectively. Mixtures were placed at room temperature for 2 h, and then transformed into 6-leave stage *Nicotiana benthamiana* by stab inoculation. After culture for 72 h at 23 ± 1 °C, fluorescence signals were observed at 400× magnification by laser confocal microscopy.

### Phloem sap collection and analysis

To demonstrate the role of the two transporters in phloem loading of l-Val-PCA, the transformant carrying recombinants pART27-RcLHT1-eGFP and pART27-RcLHT7-eGFP were cultured for 18 h in 400 mL LB medium containing 50 μg/mL spectinomycin and 20 μg/mL rifampin. After centrifugation and resuspension, the transformant was introduced into seedlings by vacuum agroinfiltration (0.09 MPa, OD600 = 1.2, 20 plus 20 min). After culturing at 18 °C in dark for 72 h, fluorescence signal strength in seedlings was surveyed under UV light. The seedlings that introduced pART27-eGFP were used as controls.

Cotyledons cultured at 18 °C in darkness were harvested, quickly frozen in liquid nitrogen and stored at − 80 °C for RNA isolation. The total RNA was extracted and the first-strand cDNA was synthesized as above. Two primers for qRT-PCR of RcLHT1 and RcLHT7 were designed using Primer Premier 5.0 software. Then the relative expression levels of *RcLHT1* and *RcLHT7* were detected after incubation for 72 h. The relative expression levels at 0 h were used as controls.

The phloem sap collection method was similar to that described by Rocher et al. [29]. The samples were analyzed by HPLC (Haineng LC7000, Jinan, China) after phloem sap was diluted with UHQ-grade water (1 + 9 v/v) and purified through a sterile filter (r = 0.22 μm). The target l-Val-PCA was separated with a C18 reversed-phase column (length 150 mm, inner diameter 4.6 mm, 5 μm). The mobile phase consisted of methanol and water containing 0.1% phosphoric acid at a flow rate of 0.8 mL/min, and the injection volume was 10 μL. Various standard solutions (0.4, 0.8, 1, 2, and 5 mg/L) of test compounds for calibration curves were prepared in methanol. Results were processed with Wookinglab software v00.02.20.00 (Haineng, Jinan, China). The data were subjected to ANOVA followed by mean separation using Dunnett’s test in SPSS software.

## Results and discussion

### Exogenous gene was expressed in *Ricinus communis* seedling

**Fig. 1:**
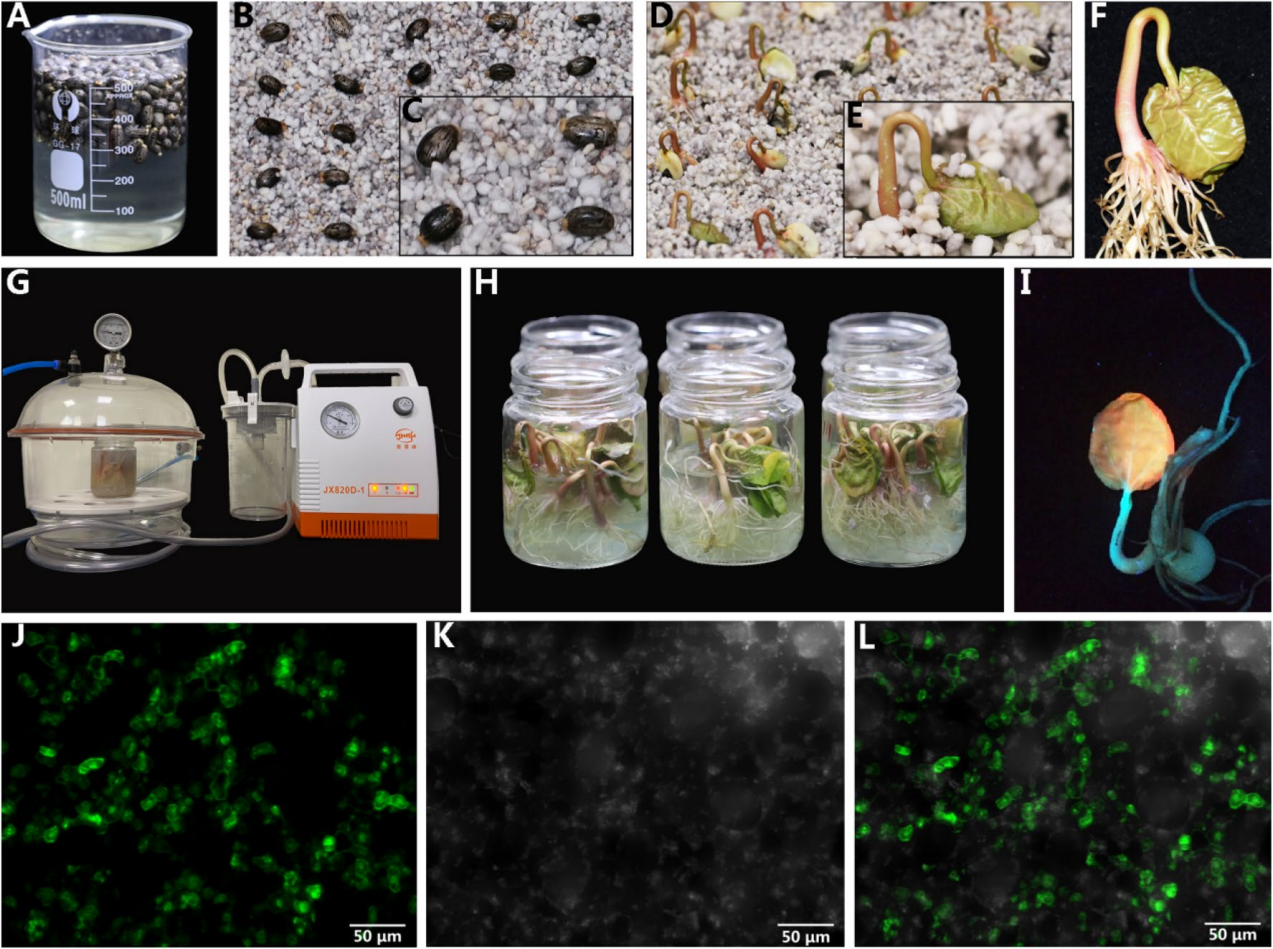
Construction of vacuum agroinfiltration in *Ricinus communis* seedling. **A** Castor bean seeds were immersed in water in 500 mL beaker for 24 h at 27 ± 1 °C. **B**, **C** Seeds were sowed in wet vermiculite. **D**, **E** Seeds were cultured while maintaining humidity for 6 days. **F** Seedlings with stems 3.0 ± 0.2 cm across, were selected and endosperm (20 mm length) carefully removed. **G** Endosperm-excised seedlings were placed in *Agrobacterium* suspension in 250 mL bottles of plant tissue culture. Vacuum infiltration was at 0.09 MPa for 20 min. **H** Seedlings were soaked in Hogland solution and cultured at 18 °C in dark for 72 h. **I** eGFP were observed under UV light. **J**–**L** The eGFP signal observed at 100× magnification by laser confocal microscopy under fluorescence, bright, and merged fields

The fluorescence signals were surveyed 72 h after pART27-eGFP was transformed into *Ricinus* seedlings. eGFP signals were observed in most seedling leave under UV light, but signal strength varied. Strong signals were commonly observed alongside the veins and weak signal were at the leaf margin (Fig. 1I). This may be due to the stomates were the main channel for vacuum infiltration of *Agrobacterium* into leaves and less stomates were found at the margin area of *Ricinus* leaves [39], and thus less *Agrobacterium* was accumulated at leaf margin. Under UV light, seedlings with strong fluorescence signals can be easily selected and used in subsequent experiments. eGFP signals were checked by laser confocal microscopy and strong fluorescence signals were observed in mesophyll cells (Fig. 1J). The outline of mesophyll cells were also observed under bright light (Fig. 1K) and overlay effect under fluorescence and bright fields were showed in Fig. 1L. Overall, as showed in Fig. 1, using the vacuum agroinfiltration strategy, an exogenous gene was successfully expressed in seedlings. Vacuum infiltration has allowed for high-efficiency introduce of the *Agrobacterium* binary vector into exposed plant tissues and high-level expression for target proteins [40], especially for the plants with hard or succulent leaves [41]. In this study, seedlings were transferred into Hogland solution after infiltration, and cultured for 72 h at 18 °C in the dark [33]. This low temperature prevented fast growth and disappearance of the hook region, which is approx. 1.0 cm apart from the cotyledons as show in Fig. 1F, I and convenient for the incubation of cotyledons, because these transformed seedlings need to be used for further collection of phloem sap. However, the effects of lower temperatures on eGFP expression need further investigation.

### Optimization of transient expression parameters

**Fig. 2:**
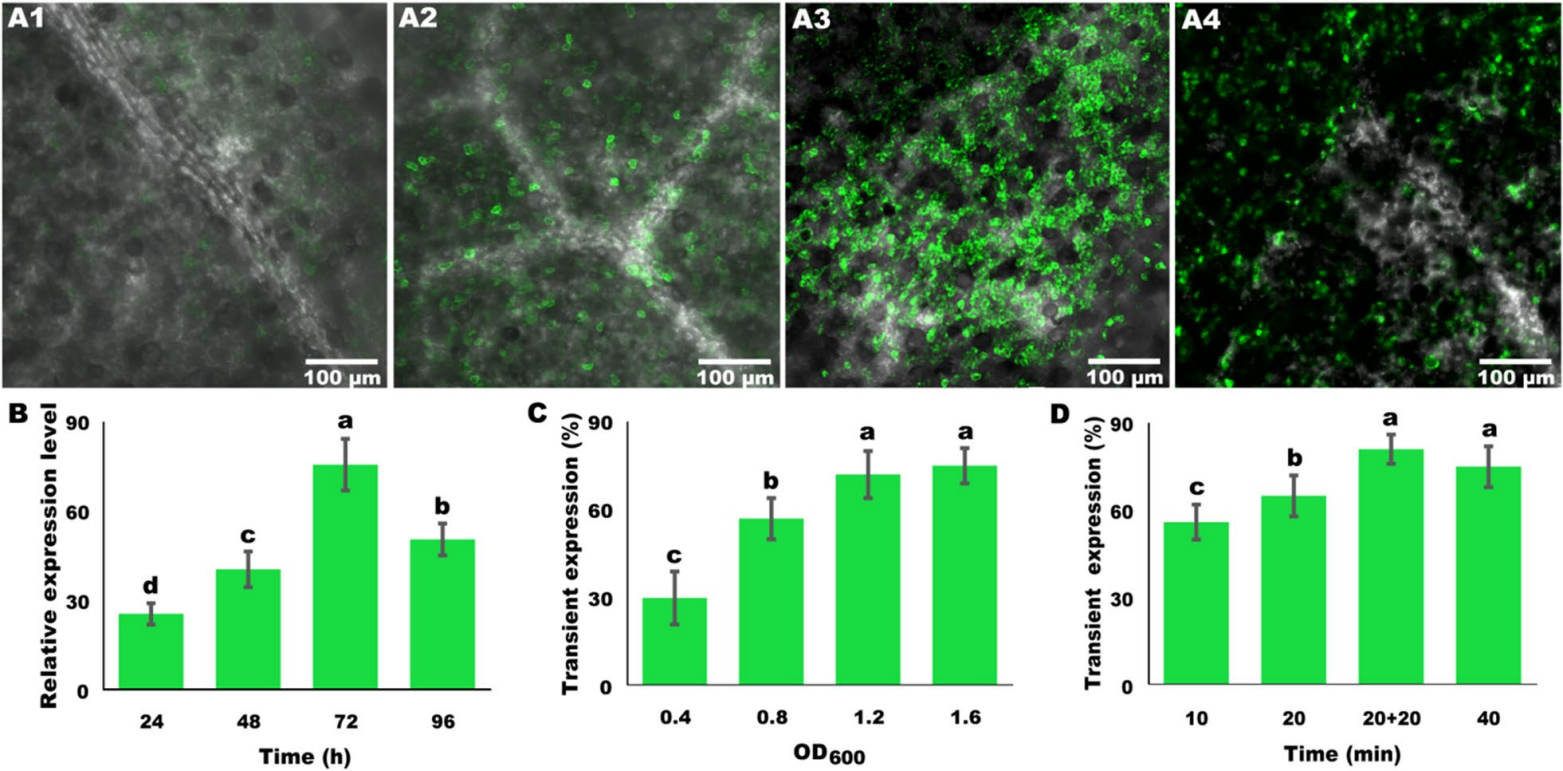
Optimization of overexpression parameters. **A1**–**A4** Observation of fluorescence strength by laser confocal microscopy in merged field at 24 h, 48 h, 72 h, and 96 h. **B** Relative expression level of *eGFP* after 24 h, 48 h, 72 h, and 96 h, detected respectively. **C** Optimization of *Agrobacterium* concentration. Series *Agrobacterium* cell densities, including OD_600_ at 0.4, 0.8, 1.2, and 1.6, were used. **D** Optimization of infiltration time. The duration of vacuum infiltration was set at 10 min, 20 min, 20 plus 20 min and 40 min. All data were collected and handled from three biological replicates. Different samples were used during these biological replicates. Letters on the graphs denote statistically significant differences (ANOVA, *P* < 0.05)

The change of fluorescence strength and eGFP gene expression level after infiltration were surveyed. Results showed that eGFP signal could be observed at 24 h, though the signal was weak; and then the fluorescence signal increased and reached peak level at 72 h, decreasing by 96 h (Fig. 2A1–A4). Consistently, the qRT-PCR analysis also confirmed that *eGFP* was continuously up-regulated from 24 to 72 h. The maximum expressed level appeared at 72 h, which was about 76-fold comparing to control (Fig. 2B). Previous studies showed that the highest level of target gene expression could be generally observed 2–3 days post infiltration, after which the expression level typically decreases [40, 42–44].

Besides, the optimization results of *Agrobacterium* cell density with OD_600_ from 0.4 to 1.6, showed that eGFP signal strength continuously increased, and the highests level of *eGFP* expression was between OD_600_ 1.2 and 1.6 which were not significantly different (Fig. 2C). Consistent with our results, Amoah et al. [28] also found that increasing the *Agrobacterium* cell density could enhance the expression of exogenous gene, which reached a peak at OD_600_ = l.5, whereas they also chose OD_600_ = 1.2 as the optimal cell density for vacuum infiltration.

The duration of vacuum was optimized. Four patterns, including 10 min, 20 min, 40 min, and 20 plus 20 min, were tested in this study. The results showed that, with the increasing of filtration time from 10 to 40 min, fluorescence strength was continuously increased. When the infiltration time was set at 20 plus 20 min, fluorescence signal strength was found to be the strongest (Fig. 2D). Previous study showed that 20 min was needed to obtain a complete infiltration with the *Agrobacterium* suspension in adult *A. thaliana* transformation [45]. Furthermore, the vacuum extracts gases from submerged plant leaves through stomata and, when the vacuum is released and pressure rapidly increases, the suspension of *Agrobacterium* is driven to leaves to replace the extracted gases [46]. Therefore, the strategy of vacuum infiltration for a period, return to atmospheric pressure, then vacuum infiltration again has been commonly used [47].

### Subcellular localization of *RcLHT1 *and *RcLHT7*

**Fig. 3:**
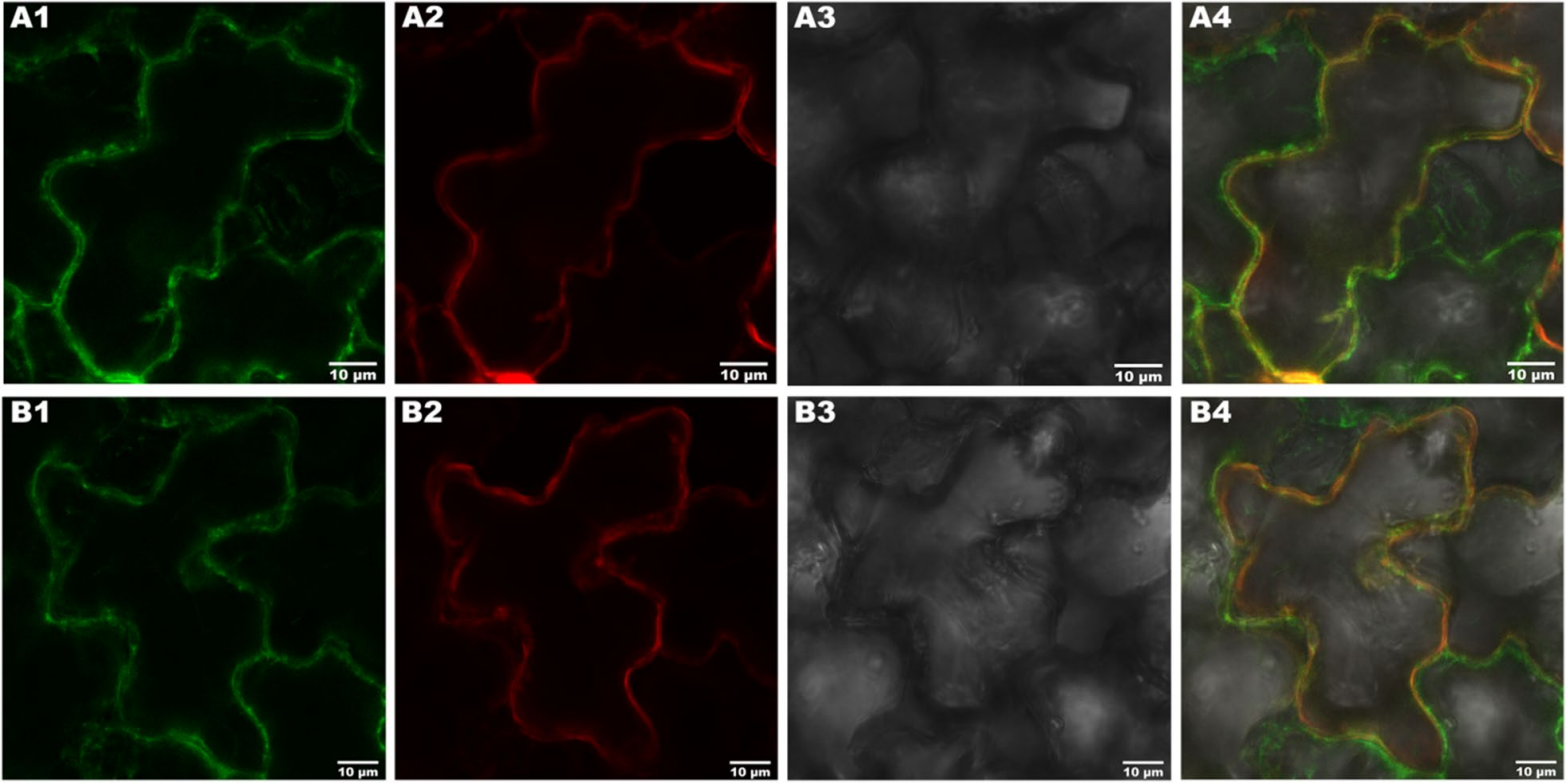
Expression and localization observation of *RcLHT1* **A** and *RcLHT7* **B** in mesophyll cells of *Nicotiana benthamiana* by laser confocal microscopy, **A1**, **B1** under green fluorescence **A2**, **B2** red fluorescence, **A3**, **B3** bright, **A4**, **B4** and merged fields

Two constructed vectors pART27-RcLHT1-eGFP and pART27-RcLHT7-eGFP with marker were co-transformed into *Nicotiana benthamiana*. After culture for 72 h, we found that the fluorescent signals of fusion proteins RcLHT1-eGFP and RcLHT7-eGFP were located in the plasma membrane of mesophyll cells (Fig. 3A1, B1). Meanwhile, as shown in Fig. 3A2, B2, the *Discosoma* red fluorescent protein (DsRed) signals of marker were also observed in the plasmalemma. Although cell shape was unclear under bright field because of the thick cuticles (Fig. 3A3, B3), we still demonstrated that two transporters, RcLHT1 and RcLHT7, are actually located in the plasma membrane under merged field (Fig. 3A4, B4). That further confirmed that RcLHT1 and RcLHT7 function as transporters and participate in membrane translocation of amino acids [48]. However, no fluorescent signals of fusion proteins RcLHT1-eGFP and RcLHT7-eGFP were found in the plasma membrane of epidermic cells.

### Overexpression of *RcLHT1 *and *RcLHT7* significantly improved the phloem mobility of l-Val-PCA

**Fig. 4:**
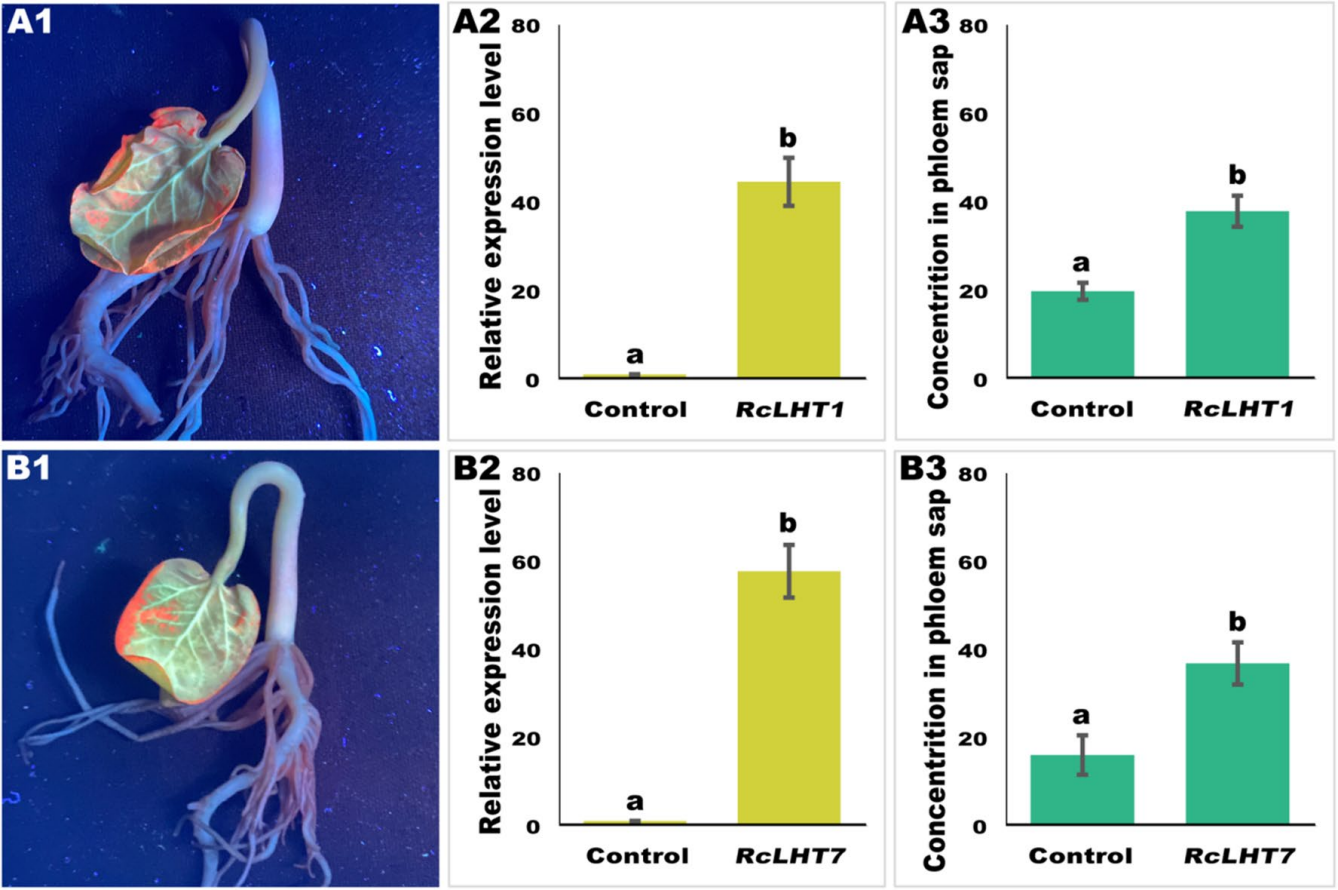
Overexpression of *RcLHT1* and *RcLHT7* improved the phloem mobility of l-Val-PCA. **A1**, **B1** UV light survey, **A2**, **B2** qRT-PCR analysis, and **A3**, **B3** measurement of l-Val-PCA concentration in phloem sap collection after overexpression of *RcLHT1* and *RcLHT7* for 72 h in *Ricinus* seedlings. Overexpression of *eGFP* was used as a check when measuring l-Val-PCA concentration in phloem sap collection. Each test contains three biological replicates. Different samples were used during these biological replicates. Letters on the graphs denote statistically significant differences (ANOVA, *P* < 0.05)

Two vectors pART27-RcLHT1-eGFP and pART27-RcLHT7-eGFP were transformed into seedlings. After incubation for 72 h, fluorescent signals of fusion proteins RcLHT1-eGFP and RcLHT7-eGFP were surveyed. As showed in Fig. 4A1, B1, similar to eGFP, strong fluorescence signal was observed alongside the *Ricinus* cotyledon veins instead of leaf margins under UV light. Meanwhile, qRT-PCR showed that the relative expression level of *RcLHT1* significantly higher than control for 43-fold (Fig. 4A2), and *RcLHT7* higher for 52-fold (Fig. 4B2). The above results indicated *RcLHT1* and *RcLHT7* acquired successful transient expressed in seedlings.

Then the loading function of RcLHT1 and RcLHT7 toward l-Val-PCA was surveyed. The standard curve (y = 0.11747x + 2.33291) was built and used for HPLC determination. The correlation coefficient was 0.998. The results of HPLC revealed that the concentration of l-Val-PCA in phloem sap was 37.85 μM after overexpression of *RcLHT1*, and the control without overexpression was 19.79 μM. The phloem loading efficiency was improved almost twofold (Fig. 4A3). As for *RcLHT7*, the concentration of l-Val-PCA was 36.82 μM, and the control without overexpression was 16.04 μM. The phloem mobility was improved 2.3-fold (Fig. 4B3). These results indicated that overexpression of *RcLHT1* and *RcLHT7* significantly improved the phloem mobility of l-Val-PCA in seedlings, which indicating their roles in recognition and phloem loading of this vectorized agrochemical. Jiang et al. [49] similarly found that *AtLHT1*, an *Arabidopsis* gene homolog to *RcLHT1* and *RcLHT7*, participated in the uptake process of an l-glutamine-fipronil conjugate (l-GlnF), and overexpression of *AtLHT1* led to 83% increase in the uptake of l-GlnF.

Recently, several assays have been used to survey the transporting functions of plasma-membrane carriers to vectorized agrochemicals. For example, *Xenopus laevis* oocytes were used to decipher the affinity of plant monosaccharide transporter, RcSTP1, to a glucose-fipronil conjugate (GTF) [13]; and *Arabidopsis thaliana* was used to study the function of AtAAP1 in promoting the rates by roots of the alanine-chlorantraniliprole conjugate [14]. Compared to above systems, the *Ricinus* seedling system can be directly used to investigate the recognition and phloem loading functions of transporters of vectorized agrochemicals, which will aid in strategic rational utilization of these plasma-membrane carriers.

## Conclusion

In this study, we successfully developed a protocol for *Agrobacterium tumefaciens*-mediated transformation of *Ricinus* seedling and optimized the parameters. The optimum time for detection of overexpression of exogenous gene was 72 h. The *Agrobacterium* cell density was OD_600_ = 1.2, and the infiltration time was 20 plus 20 min (vacuum infiltration for 20 min, then return to atmospheric pressure, and then infiltration for another 20 min). Using this method, overexpression of *RcLHT1* and *RcLHT7* can significantly increase the phloem translocation of l-Val-PCA in sieve tubes of seedlings, suggesting that RcLHT1 and RcLHT7 participate in the recognition and phloem loading of l-Val-PCA. Our results provide a foundation for researchers to directly investigate the loading functions of plasma-membrane transporters toward vectorized agrochemicals in *Ricinus* seedling system.

## Data Availability

All data and material generated or analysed during this study are included in this published article.
